# Correlative imaging of ferroelectric domain walls

**DOI:** 10.1038/s41598-021-04166-y

**Published:** 2022-01-07

**Authors:** Iaroslav Gaponenko, Salia Cherifi-Hertel, Ulises Acevedo-Salas, Nazanin Bassiri-Gharb, Patrycja Paruch

**Affiliations:** 1 Department of Quantum Matter Physics, University of Geneva, 1211 Geneva, Switzerland; 2 G.W. Woodruff School of Mechanical Engineering, Georgia Institute of Technology, Atlanta, GA 30332 USA; 3 CNRS, Institut de Physique et Chimie des Matériaux de Strasbourg, UMR 7504, Université de Strasbourg, 67000 Strasbourg, France; 4 School of Materials Science and Engineering, Georgia Institute of Technology, Atlanta, GA 30332 USA

**Keywords:** Atomic force microscopy, Polarization microscopy, Scanning probe microscopy, Nonlinear optics, Ferroelectrics and multiferroics, Computational science, Scientific data, Software

## Abstract

The wealth of properties in functional materials at the nanoscale has attracted tremendous interest over the last decades, spurring the development of ever more precise and ingenious characterization techniques. In ferroelectrics, for instance, scanning probe microscopy based techniques have been used in conjunction with advanced optical methods to probe the structure and properties of nanoscale domain walls, revealing complex behaviours such as chirality, electronic conduction or localised modulation of mechanical response. However, due to the different nature of the characterization methods, only limited and indirect correlation has been achieved between them, even when the same spatial areas were probed. Here, we propose a fast and unbiased analysis method for heterogeneous spatial data sets, enabling quantitative correlative multi-technique studies of functional materials. The method, based on a combination of data stacking, distortion correction, and machine learning, enables a precise mesoscale analysis. When applied to a data set containing scanning probe microscopy piezoresponse and second harmonic generation polarimetry measurements, our workflow reveals behaviours that could not be seen by usual manual analysis, and the origin of which is only explainable by using the quantitative correlation between the two data sets.

## Introduction

Technological advances rely heavily on the development of new materials, both in terms of understanding their fundamental properties and through the engineering of their composition and structure. In the last decades, complex oxide materials have shown promise in photovoltaics applications^1^, high efficiency actuation and sensing^2^, as well as information storage^3^. Among these materials, ferroelectrics are of particular interest. They possess a spontaneous polarization switchable under the application of an electric field, and a wealth of associated functional properties including strong nonlinear electro-optical effects, very high piezo- and pyroelectric responses, and in some cases magnetoelectric coupling^4^. Recently, the focus has shifted towards the investigation of ferroelectric domains and domain walls—interfaces separating regions of differing polarization orientation—as their intrinsically nanoscale nature and unique functional properties make them potentially useful for device applications^5,6^.

Ever more detailed studies have revealed unusual behaviours such as localized electrical conduction^7,8^, mechanical shear^9^ or magnetic ordering closely tied to the distinct structure of domain walls^10^. Whilst most of these investigations were carried out using a combination of high resolution scanning probe microscopy (SPM) based techniques^11^ to explore the electromechanical and electrochemical responses, optical approaches—in particular second harmonic generation (SHG) microscopy with polarimetry analysis—are increasingly being used as a way to non-invasively probe the internal structure, chirality and polarization of domain walls^12–16^.

Combining the two felicitously complementary techniques—with sequential SPM and SHG measurements of the same intrinsic or engineered domain structure—provides an opportunity to extract a complete structural and functional portrait of domain walls. Indeed, such multi-technique studies have already been leveraged at varying length scales and with a varying degree of correlation. At the most basic level, SPM imaging has been used in order to locate areas of interest for coarser resolution micro-Raman measurements, enabling a better understanding of the resulting signal from the qualitative model of the underlying domain structrures^17^. As the spatial resolution of optical techniques increased, joint usage of optical and SPM techniques allowed the gathering of a wealth of knowledge about the structure, hierarchy and organisation of ferroelectric domains, as well as their properties in uniaxial ferroelectrics such as lithium niobate and tantalate^18,19^. Overall, such multifaceted approaches are expected to become the norm in the community, in particular due to the development of ever more precise spatially resolved functional measurement techniques, allowing the probing of a large variety of electronic and optical phenhomena at the nanoscale resolutions^20^.

However, despite their complementarity and successful application at submicron lengthscales, the very different nature of the various optical and SPM techniques has so far made a fully quantitative and correlative analysis of their observables at the level of subnanometric domain walls extremely challenging. Specifically, in SHG polarimetry experiments, stacks of images are recorded as a function of the laser polarization and the analyzer angle, with multiple potential contributors combining into the resulting SHG signal and making analysis and interpretation highly nontrivial^21^, in particular in ferroelectric and multiferroic thin films^22–24^. As a result, disentangling the SHG signal of a specific domain wall from the response arising from neighboring walls, adjacent domains, and surface/interface (background) contributions can be an extremely tedious task, made more so by the very small size of the investigated objects (far below the resolution limit of optical methods). In contrast, SPM based techniques possess a high spatial resolution and can be used to directly probe the polarization orientation, mechanical response, surface charge dynamics, electrical conduction and other surface properties. However, SPM is prone to artifacts such as signal contributions from electrochemical and electrostatic interactions, or mechanical cross-talk, which pose challenges for the quantitative and sometimes even qualitative interpretation of the ferroelectric polarization. Above all, the very different observation scales and analysis approaches make a quantitative correlation between SHG and SPM datasets a formidable problem.

A solution to this challenge is provided by the recent advances in the use of computer vision and machine learning for the investigation of ferroelectric materials^25–28^. Computer vision can be used to match data based on common features across the two completely different techniques^29^. Machine learning techniques can not only identify and quantify behaviours in large data sets across multiple parameters, but through additional pre-processing methods such as dimensional stacking can provide physical insights into the intrinsic correlations at multiple length scales and across characterization methods^30^.

In this paper, we leverage a combination of machine learning based techniques with data stacking approaches in order to enable a fast, correlative, and unbiased analysis of SHG and SPM data sets. This correlative approach is first introduced and discussed from the workflow point of view, then demonstrated via a comparative study of the different data analysis methods. Based on a data set acquired on a Pb($\hbox {Zr}_{0.2}\hbox {Ti}_{0.8}$)$\hbox {O}_3$ thin film, the analysis aims to disentangle the different origins of the overall SHG polarimetry response, specifically at ferroelectric domain walls delimiting triangular *c*-domains. Classical analysis methods—including manual data extraction and superpixel analysis—are presented, and compared to K-means clustering on the stacked SHG dataset, which is not only consistent with the previously published manual analysis^15,16^, but provides a significant improvement in terms of identifying physically meaningful and distinct behaviours. This stacked K-means clustering analysis—requiring no manual input other than number of behaviours—greatly enhances the ability to analyze SHG signals, especially in more complex cases with the presence of a background signal and/or combination of multiple physical behaviours. In particular, it allows us to identify additional behaviours revealed by machine learning that might have been discarded as “noise” or “experimental error” during manual analysis. Finally, we extend this approach to include both the SHG signal and co-localized PFM response through spatially correlative machine learning—enabling a better discrimination of domain and domain wall functional responses, and a truly nanoscale understanding of their structure-property relationships.

## Correlative analysis workflow

The key paradigm behind the proposed workflow is that of a data-driven analysis approach. Both SHG and SPM data acquisition produce two-dimensional maps that are spatially referenced—and can therefore be directly compared after an appropriate coordinate map transformation^29^. Once generated, the corrected observable maps can be stacked into a single aggregated dataset, where each spatial point or voxel contains a heterogeneous vector combining the parameter space of both techniques. The stacking itself enables correlative analysis to be performed, allowing known physical and chemical constraints to be taken into account^30^. The individual steps of the correlative analysis workflow are discussed below.

### Distortion correction

The spatial cross-referencing of the different measurements is performed through distortion correction based on image registration methods, and is involved in two major steps of the complete analysis workflow. In both cases, the *findTransformECC* function from the OpenCV3 library is used, finding a geometric transform between two images based on intensities using the enhanced correlation coefficient maximization method^31^.

First, we address instrumental and thermal drift resulting from sequential SHG acquisition performed for each analyzer/polarizer configuration. The drift is corrected through the use of a translation geometric transform (*cv2.MOTION_TRANSLATE*), performed on each consecutive pair of SHG images which have been subjected to a median subtraction and a modulus operation. The generated list of cumulative offsets is then applied to the original SHG data, yielding a position-corrected SHG dataset.

Second, the corresponding SPM images are matched to the SHG dataset. As the images were acquired through piezoresponse force microscopy, not only surface topography but also the phase and amplitude of the sample electromechanical response are available. The latter represent the qualitative orientation and magnitude of the polarization. As the matching has to be performed based on information common to both datasets, proxy images have to be generated to allow comparison between them. For the SHG dataset, the median of the stacked and corrected dataset modulus is used, highlighting the position of the ferroelectric domain walls with a width characteristic of the optical resolution of the technique. For the SPM dataset, the phase representing the polarization orientation is binarized, then cleaned to filter small holes/objects, and finally eroded/dilated to provide a precise position of the domain wall. The final domain wall signal is then Gaussian-blurred to the spatial equivalent of the optical resolution. The resulting SPM proxy is further distortion-corrected to the SHG proxy through the use of a homography transformation (*cv2.MOTION_HOMOGRAPHY*), as more than simple translation shifts may be involved in the use of heterogeneous data.

### Data stacking

Once all data have been corrected to have the same spatial coordinates, it can be analyzed in a correlative manner through the use of data stacking^30^. As each spatial location is associated to a vector containing the response at each analyzer/polarizer configuration, as well as the phase and amplitude of the piezoresponse force microscopy, any computation simultaneously involving more than one of these voxels—or measurement conditions—will inherently induce a spatial correlation within the results.

For instance, one can subject only the SHG analyzer/polarizer elements of the voxel to further analysis, enabling the computation and demixing of polar plots at each location of the measured area. This is the standard SHG analysis process, and is usually done manually through the selection of regions of interest and averaging in individual images. This process is discussed in more detail below, clearly showing the advantages of data stacking and correlative analysis workflow over the more traditional methods.

There are fundamentally no limitations to what data can or should be stacked together for analysis, as long as these share common parameters. For instance, multiple SHG data sets acquired with the same analyzer/polarizer pairs could be stacked together and directly compared, or other spatially resolved techniques could be used to acquire different physical observables on the same area. The SPM images are one such example, as they were acquired on the same set of domains prior to the SHG measurements. Thus, extending the observable vector at each location with the SPM data enables us to include the electromechanical response in further analyses, rendering this workflow truly correlative.

### Machine learning analysis

In the presence of large or heterogeneous datasets such as described above, physical intuition might not be sufficient to grasp the underlying physics or to segment the data in an unbiased manner. A path forward is provided by machine learning based techniques, which can be used for the purpose of data regression, classification, dimensional reduction, clustering, or association^32^. Amongst these, clustering and dimensional reduction have been the most widely used as tools to objectively reveal underlying behaviours in materials science datasets.

In the present analysis, clustering was performed through a K-means algorithm following the subtraction of the mean background signal of each SHG image in order to segment the SHG dataset into regions of interest with distinct behaviors. Similar analysis is usually performed by manually and arbitrarily delimiting the regions of interest within SHG maps. With K-means, however, an objective Euclidean distance criterion is used to segment the dataset into spatially indexed clusters, with centroids encoding the differing mean behaviours within each cluster.

Although not within the scope of this work, more advanced machine learning dimensional reduction techniques could also be applied to the complete dataset, supplementing the workflow. These include principal component analysis, independent component analysis or non-negative matrix factorization. The resulting decompositions will thus enable spatially co-located and intermixed behaviours to be separated based on various mathematical criteria. The use of these approaches is however subject to caution, as more complex underlying assumptions may render the physical interpretation more challenging^33^.

### Workflow implementation

The complete correlated analysis workflow presented in this paper is implemented in a standalone Jupyter notebook using the Python 3 programming language. The notebook contains code to load and preprocess the data files, to correct for distortion, and to align both SHG and SPM datasets, as well as the K-means analysis code. Additionally, all functions for the workflow are implemented in Hystorian, a Python library focused on data processing traceability, reproducibility, and workflow history archival^34^.

## Results and discussion

### Data set

In this study, we investigate the internal structure of nominally uncharged $180^\circ$ ferroelectric domain walls in a tetragonal PbZr($_{0.2}\hbox {Ti}_{0.8}$)$\hbox {O}_3$ (PZT) thin film, grown on top of an $\hbox {SrTiO}_3$ Ti-terminated substrate with an intercalated $\hbox {SrRuO}_3$ back electrode. The resulting film polarization is uniaxial, out-of-plane, and monodomain up-oriented. Artificial down-oriented *c*-domains are created by means of a biased scanning conductive AFM tip, with nanoscale domain walls separating the domains of opposite polarization orientation. As we have previously demonstrated, SHG can be used to reveal the non-Ising nature of these domain walls^15^.

**Figure 1:**
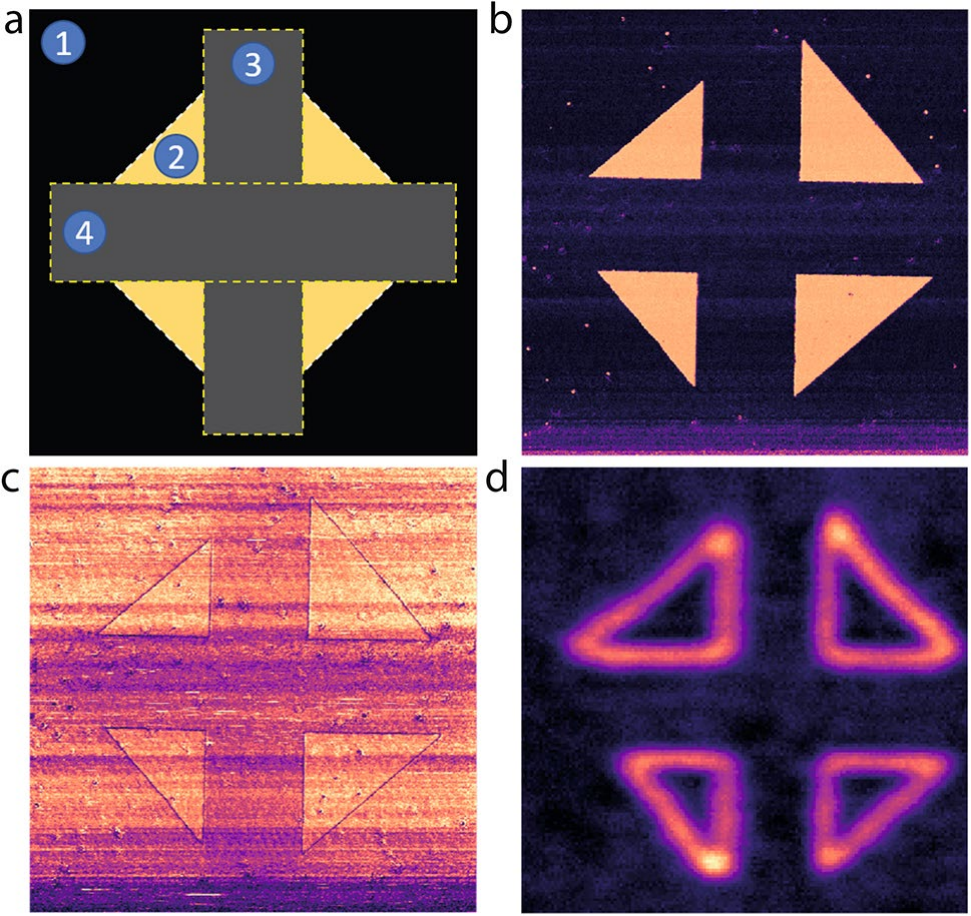
Sample domain structure. (**a**) Polarization switching schematic of the written domain configuration. The film is originally fully up-polarized (1). First, a $45^\circ$ square (2) is patterned by applying a positive voltage to the up-oriented thin film of PZT using a scanning probe microscopy tip. Then, two perpendicular stripes at $0^\circ$ and $90^\circ$ (3, 4) are written with negative voltage to reverse the polarization to the as-grown down-oriented state. The four resulting triangle domains are imaged both via PFM (**b**) phase and (**c**) amplitude, and (**d**) SHG. The latter was acquired at an angle of $90^\circ$ with respect to the PFM due to the experimental configuration.

The domain structure under investigation consists of four right angle triangles, engineered to provide a set of vertical, horizontal and oblique domain walls. It is generated through a series of bias-induced polarization reversals during scanning. First, a 45$^\circ$ square is switched by applying a positive voltage to the up-oriented thin film of PZT. Then, two perpendicular stripes at 0$^\circ$ and 90$^\circ$, respectively, are written with negative voltage to switch the polarization back to the original down-oriented state. This results in the four down-polarized triangles shown in Fig. 1a. The corresponding piezoresponse force microscopy (PFM) phase and amplitude images in Fig. 1b,c demonstrate a full polarization reversal within the target triangles. An isotropic SHG intensity image is shown in Fig. 1d, indicating that the SHG signal comes almost exclusively from the domain walls. A $90^\circ$ angle is present between the two data sets due to the difference in experimental configuration, and has no effect on the subsequent analysis.

### Artisanal SHG segmentation methods

#### Traditional method

**Figure 2:**
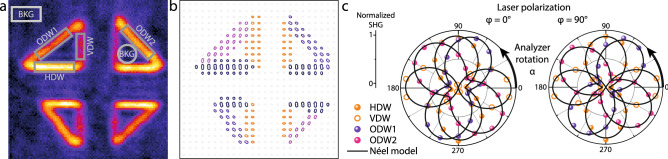
SHG polarimetry analysis methods. (**a**) Isotropic SHG image (laser polarization angle $\varphi =0^\circ$, without analyzer) showing five different regions of interest corresponding to horizontal and vertical domains walls (labeled HDW and VDWs, respectively) as well as background (BKG), and oblique domain walls (ODW1,2). (**b**) Polarimetry mapping is obtained by subdividing each image of the dataset into superpixels containing $4 \times 4$ pixels. For each superpixel, a polar plot is defined, describing the local variation of the SHG signal as a function of the analyzer angle at a given fundamental wave polarization ($\varphi =0^\circ$ in this case). The local SHG intensity variations in each DW derived from the manual segmentation method is given in (**c**) for both analyzer/polarizer configurations. In these plots, the averaged background signal was subtracted.

The isotropic SHG image reveals an increased intensity at the domain walls, as shown in Fig. 2a. As the domain walls of different physical orientation are expected to give a different polar response, we start by averaging regions of similar behaviour.

Through our existing knowledge and physical insight, we can postulate the existence of five independent groups of SHG emitters (objects) within the written triangular down-polarized domains. These are visually identified in Fig. 2 as (a) the background (BKG), horizontal domain walls (HDWs), vertical domain walls (VDWs), as well as oblique domain walls with positive (ODW1) and negative (ODW2) tilt angles. This is the traditional way of tracking objects for SHG polarimetry measurements: the position of the object is identified within a sequence of analyzer/polarized configurations and changes in its intensity are followed through the manual selection of different regions of interest (ROI), with each ROI containing an individual object (e.g., one specific type of domain wall). This process enables us to build the polar response of each object by extracting and averaging the SHG intensity at each analyzer/polarized configuration. The resulting polar plot for this traditional manual extraction method of the five different regions is shown in Fig. 2c, demonstrating good agreement with a Néel model for the domain wall internal structure for all four domain wall groups.

#### Superpixel averaging method

As an alternative method, each image can be subdivided into a checkerboard consisting of homogeneous squares of multiple pixels in which the intensity is averaged to enhance the signal-to-noise ratio. The process is repeated for each analyzer/polarized configuration, yielding a separate polar plot for each of these superpixels. All of the polar plots are then represented at their superpixel center position, yielding a plot field like the one shown in Fig. 2b. The specific shape of the resulting polar plots is analyzed visually, with similar plots in terms of intensity and anisotropy grouped as in the traditional method above, and represented in a specific color.

#### Challenges of artisanal methods

Accessing the internal structure of a domain wall with SHG polarimetry analysis requires a detailed modeling of the SHG emission based on local symmetry and polarization orientation (see “Methods”). This modeling procedure is particularly difficult in the case of thin ferroelectric films due to the contribution of the background signals, and the nanoscale nature of the domain wall itself. Indeed, even if the SHG signal arising from symmetry breaking at surfaces and interfaces is known to be small, its contribution in thin films and multilayers can often induce an anisotropic response, which could for instance result in artificially elongated polar plots. Therefore, the subtraction of the surface SHG contribution from the signal emitted by the selected ROIs can strongly alter the resulting anisotropy of the object, i.e., the polarimetry response of a domain wall in the case of this study (see Supplementary Discussion 1).

The human eye is clearly capable of detecting changes across multiple objects, but this detection is restricted to a limited number of objects at the same time. Moreover, apart from being time-consuming, purely visual separation can lead to errors related to both the vision (field of view, peripheral vision, etc) and attention (focus on specific objects) of the researcher making the decisions. The two methods presented above are clear examples of this human-machine barrier. In the traditional method, manual delimiting of the ROIs introduces a potential bias—usually favoring regions of high and uniform intensity. The superpixel analysis, in contrast, will favor the selection of larger ROIs due to the visual similarity of polar plots appearing less important than their intensity alone.

Automating data segmentation is therefore indispensable if errors arising from human intervention are to be reduced. A partial improvement is provided by standard image processing software, which allows for a semi-artisanal segmentation, generally based on histogram analysis and thresholding. This method provides satisfactory results in unambiguous cases, where the image contains objects of discrete gray levels and an almost perfectly homogeneous background. However, it cannot be properly applied to complex systems such as the local polarimetry analysis presented here. Domain walls with different orientations superimposed onto an inhomogeneous optical response of the background (see Supplementary Fig. 1) generate a complex SHG signal, and therefore, more advanced methods such as machine learning-based clustering should be better adapted in this case.

### K-means clustering in domain wall SHG studies

**Figure 3:**
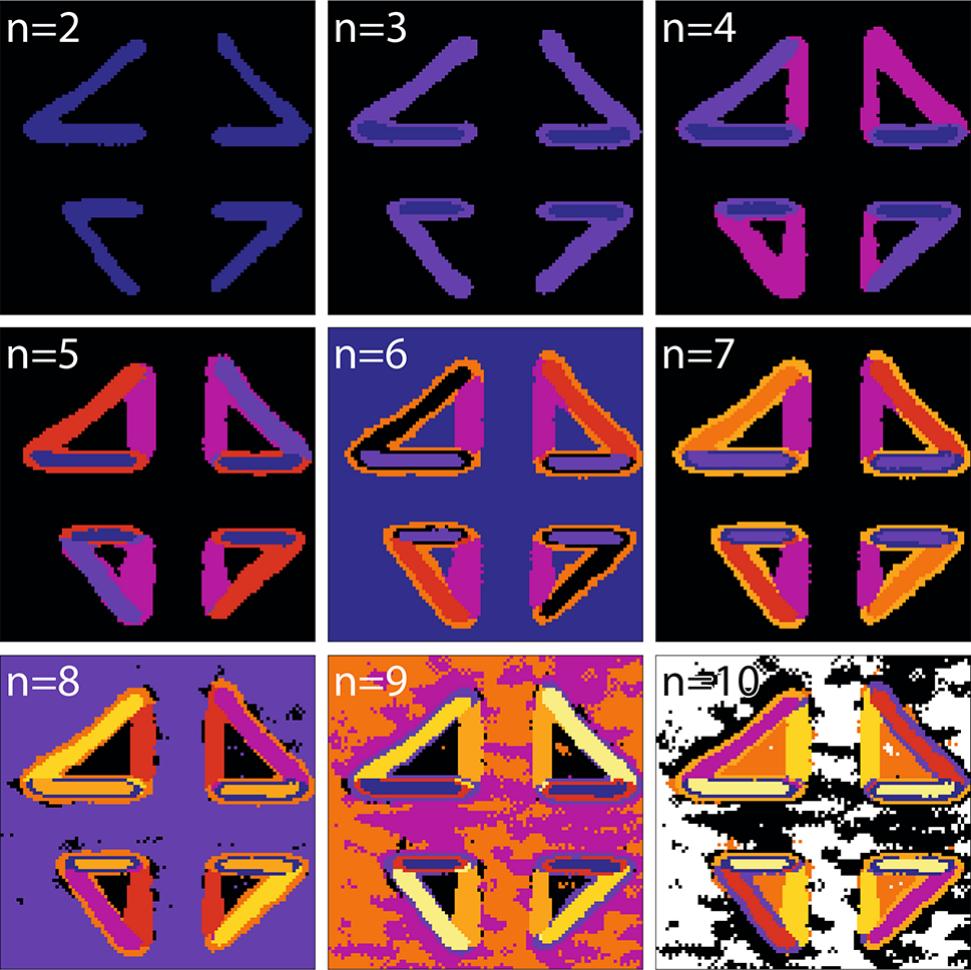
K-means clustering applied to domain wall SHG polarimetry analysis. Each image represents the spatial distribution of clusters, identified each by a differently colored region, found in the same SHG micrograph (field of view 15 × 15 μm^2^) with the analysis repeated with the number of clusters *n* varying from $n=2$ to $n=10$. As the number of clusters is increased, progressively more details are resolved as distinct in the corresponding spatial maps.

Clustering methods are particularly suited for the identification of groups with distinct properties in a given data set, based on a concept of similarity between elements within each cluster. In K-means clustering, this similarity is understood as the Euclidean distance between data points within their multidimensional parameter space. When applied to the SHG polarimetry data set, each analyzer/polarizer configuration is considered as one parameter dimension, with the complete data set represented by spatially referenced parameter space voxels. To use K-means clustering, one has to define *n*, the number of clusters. The algorithm is then initialized with *n* randomly distributed centroids (cluster centers), and each point is attributed to the cluster of the closest centroid. The centroids are then displaced to the resulting cluster center. This expectation-maximization process is repeated until convergence, with the cluster number for each parameter space vector assigned to its spatial position. The K-means analysis of the SHG polarimetry data set is shown in Fig. 3, with the number of clusters from $n=2$ to $n=10$. At the lowest cluster count, $n=2$, the resulting map displays two color-coded clusters: one containing the horizontal and the oblique walls, and the other containing the rest of the image, with background and vertical DWs. This result is obviously not satisfactory since it does not allow us to correctly discriminate walls with different geometries from the background signal. Increasing the number of clusters *n* has the effect of identifying a larger number of regions with a distinct SHG signal, with $n=5$ yielding a cluster distribution that corresponds to those identified by eye in the artisanal approaches above. Indeed, the five clusters (VDW, HDWs, ODW1, ODW2 and BKG) are fully recovered, without requiring any manual input except the number of clusters.

**Figure 4:**
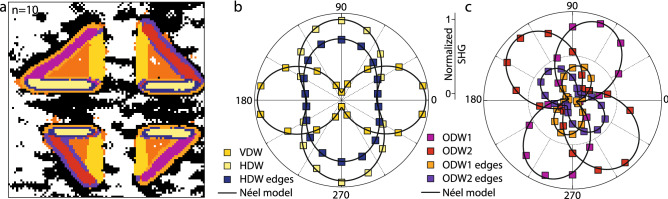
(**a**) Clustering for $n=10$, with the corresponding cluster centroids yielding polar plots in (**b**, **c**) showing SHG intensity variation with the analyzer angle at laser polarization angle $\varphi =0^\circ$. All the data are well fitted with a Néel model. Several significant features including heterogeneous background response and distinct signals at DW edges and junctions are revealed with respect to clustering with $n=5$ or traditional analysis.

Besides enabling a full automation of the image segmentation, K-means clustering gives us the possibility to resolve additional features that are impossible to identify by the human eye, simply by increasing the number of clusters *n*. These could for instance be small regions with similar polar plot behaviors throughout the analyzer/polarizer parameter space. These features are shown, for $n=10$, in Fig. 4, alongside the polar plots obtained from cluster centroids for a laser polarization oriented along HDWs. All the polar plots can be fitted with a Néel model, indicating that the internal structure of domain walls is indeed Néel type. However, a slight change of the polar plot anisotropy is present at the edges of the domain wall regions with respect to their inner cores, as can be seen clearly in Fig. 4c. The origin of such distinct interface regions is yet unclear, and may originate from different sources such as a possible mixing between the signals of domain walls and surrounding domains—or from the preferential segregation of defects, known to be favoured at the position of domain walls^8,35^. Moreover, the clustering seems to present distinct features right at the edges where the domain walls along the different directions meet and have to adapt crystallographically. Such features may stem from a complex domain structure, and would require supplementary atomistic resolution studies, for instance with transmission electron microscopy in order to be fully understood. Additionally, we can clearly identify heterogeneities within the background regions, as well as a noticeable difference between the up-polarized $c+$ and down-polarized $c-$ domain signals. Such differences are not surprising and can come either from the variations in the underlying disorder landscape of the films^36^ for the former, and from the structural modifications and strains generated through the writing of ferroelectric domain structures with an SPM tip^37^ for the latter. Overall, the additional features can be explained through physical considerations, and reveal behaviours that were previously overlooked as a result of the manual selection process in the artisanal segmentation methods.

One can naturally question the pertinence of increasing the *n* number above this value. The benefit of additional feature detection with increasing *n* must be balanced against the risk of over-fitting, where an excessively large number of clusters could lead to their noise-induced separation, while in reality they belong to a group with the same physical properties. In fact, the absolute limit of *n* is, trivially and meaninglessly, the number of data points provided to the algorithm—with each data point occupying its cluster. Thus, to answer the question of how many clusters are enough, one has to make use of physical intuition as well as quantitative metrics such as silhouette score and plots^38^. An example of such an analysis is detailed in Supplementary Fig. 4, showing that $n=5$ and $n=10$ are reasonable choices, as they lie near the beginning of silhouette score plateaus at the feature-defining cluster numbers.

### Towards cross-technique correlative analysis

As discussed above, the overarching goal of this work is to devise an approach to perform a quantitative and correlative analysis of SHG and SPM data sets acquired over the same regions. This is enabled through the use of a combination of computer vision distortion correction, machine learning based techniques,and data stacking approaches.

**Figure 5:**
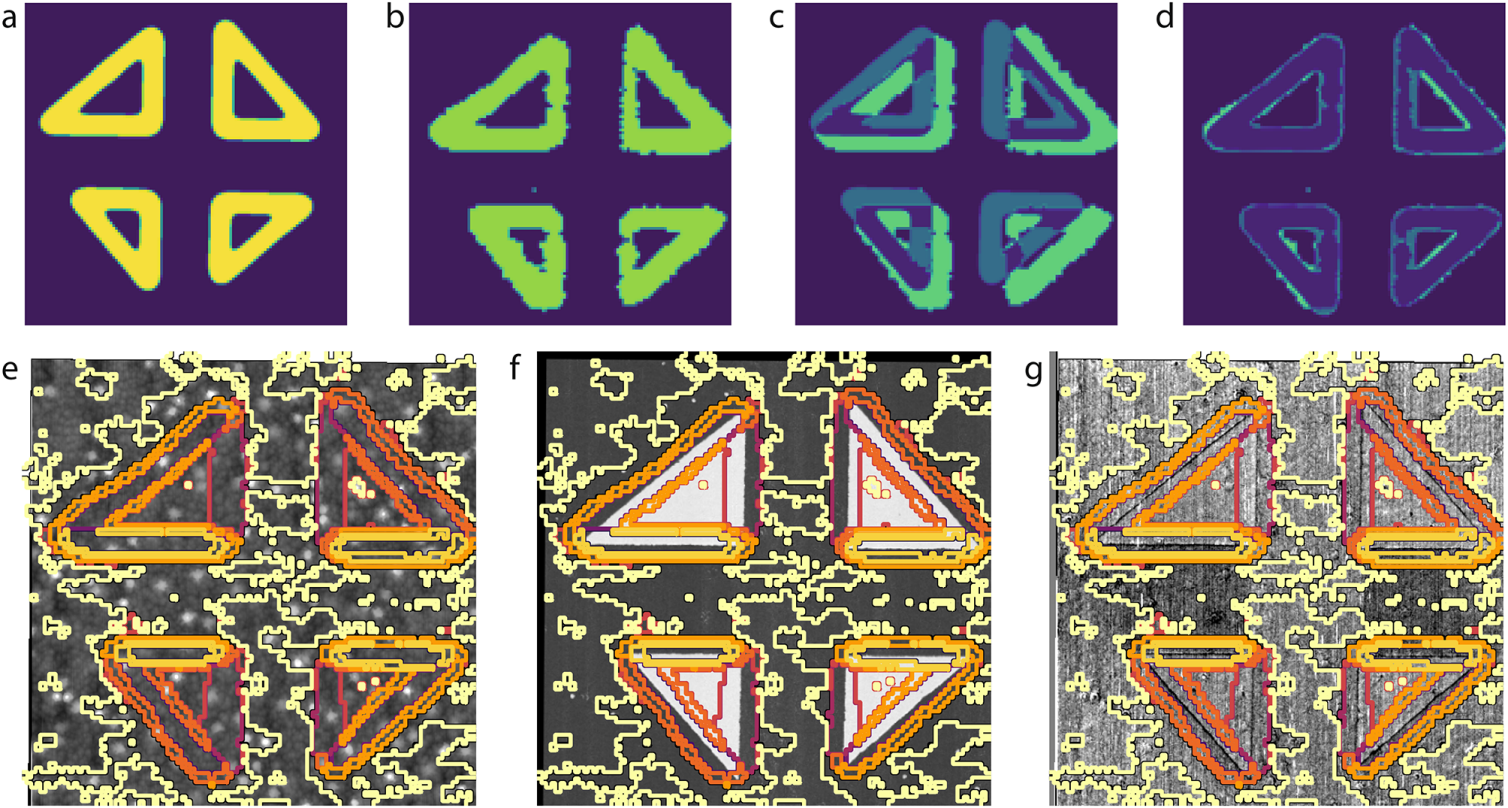
Correlative analysis of the SPM and SHG datasets. (**a**) Binarized SPM domain wall proxy image generated from the piezoresponse force micrscoscopy phase image. (**b**) Binarized isotropic SHG image. (**c**) The SPM domain wall proxy subtracted from the isotropic SHG image shows that there is a clear misalignment. (**d**) Once aligned through the use of computer vision distortion correction, there is a quasi-perfect spatial match between the SHG and SPM data sets. This enables a quantitative correlative analysis, for instance the comparison of the features in $n=10$ clustering (colour lines indicating cluster borders) and the (**e**) topography, (**f**) PFM phase or (**g**) PFM amplitude.

The correlation of the two data sets is performed on the spatial dimensions, as both SPM and SHG are spatially resolved techniques. However, not only the length scales but also the spatial resolution of these techniques are radically different. Whereas SPM is capable of nanometer-scale precision, SHG is limited by the optical wavelength of the laser beam. It is thus necessary to generate a proxy image from the SPM data set to perform spatial correlation with SHG. To this end, the piezoresponse phase—allowing a clear identification of the ferroelectric domains—is first binarized and then skeletized through dilation and erosion, yielding a pixel-wide domain wall image. The image is then Gaussian-blurred to the resolution of SHG, finally resulting in the proxy image shown in Fig. 5a. The isotropic SHG image is then binarized, as shown in Fig. 5b, and both images are subjected to a computer vision based image registration algorithm based on the effective correlation coefficient method and a homography matrix transformation^29,31,39^. The difference between the proxy and SHG images is shown in Fig. 5c,d, respectively before and after the image registration. A quasi-perfect spatial match is established between both data sets through the resulting homography transformation.

Once registered, the SPM data can for example be used to understand the origin of the various optical responses found through the K-means clustering discussed above. Here, the $n=10$ cluster image is overlaid in Fig. 5e–g on the surface topography, piezoresponse phase, and piezoresponse amplitude, respectively. We note that the image registration performed admirably, and all clusters representing the domain wall cores are well aligned with the domains as imaged by SPM. Secondly, the variations in the SHG background signal largely correlate with the numerous island-like topographic features present on the surface. This is not unexpected, as SHG is highly sensitive to surface and interface effects. However, we note that this effect is quite small, as the splitting of the background into two clusters occurs at a high cluster number, $n=9$, meaning the difference in the measured polar plots is subtle.

Once combined, the two SPM and SHG sets can be treated together in order to further extract correlations across the different length scales. The above analysis can for example be extended by the inclusion of the SPM channels as additional parameter dimensions in a full K-means analysis, as illustrated in the Supplementary Note 3.

## Conclusion

We have demonstrated a workflow for the automated recovery of SHG features through the use of machine learning and data stacking techniques. By applying the workflow on a previously published data set, we have unearthed significant additional insights into the structure-property relationships and functional responses at domain walls in PZT thin films, with much more limited intervention and effort. By combining the workflow with computer vision approaches, we have presented a quantitative correlative analysis of SHG and SPM data sets—two techniques with radically different modes of operation and spatial resolutions.

*In fine*, the approach presented here opens up new possibilities at the cross-roads between materials physics and data science. We envision that in the future, a use of holistic, cross-technique analysis will become standard practice in the quest for the understanding of material physics. The correlative workflow discussed in the current work is driven by data science, and is applicable to any set of spatially resolved techniques. This opens a wide range of possibilities for correlated studies of complex oxides or mixed phase polymers by a combination of optical, magnetic, morphological, and functional probing techniques.

## Methods

### Second-harmonic generation microscopy

The SHG measurements were conducted with a scanning confocal microscope in reflection geometry. In order to analyze the internal structure of the domain walls, polarimetry experiments and simulations based on symmetry arguments are conducted. The modeling of the SHG polarimetry response is based on the analytic form of the SHG by assuming a non-Ising character (i.e., Bloch or Néel type structure) of the domain walls. In the case of tetragonal Pb($\hbox {Zr}_{0.2}\hbox {Ti}_{0.8}$)$\hbox {O}_3$ films, the data are well fitted assuming *m* point group symmetry, which corresponds to a Néel-type domain wall structure. A detailed description of the method can be found in^16^.

### Scanning probe microscopy

The scanning probe microscopy measurements were performed in piezoresponse force microscopy mode on a Bruker Dimension 3100 atomic force microscope. The details of the measurement parameters may be found in^15^.

## Supplementary Information

Supplementary Information.

## Data Availability

Data and analysis code is available at the long term storage Yareta project repository, hosted at the University of Geneva^40^.

## References

[1] Almora O (2020). Device performance of emerging photovoltaic materials (version 1). Adv. Energy Mater..

[2] Wilson SA (2007). New materials for micro-scale sensors and actuators. Mater. Sci. Eng. R. Rep..

[3] Cao Q (2020). Nonvolatile multistates memories for high-density data storage. ACS Appl. Mater. Interfaces.

[4] Whatmore R (2017). Ferroelectric Materials.

[5] Catalan G, Seidel J, Ramesh R, Scott JF (2012). Domain wall nanoelectronics. Rev. Mod. Phys..

[6] Meier D, Seidel J, Gregg M, Ramesh R (2020). Domain Walls: From Fundamental Properties to Nanotechnology.

[7] Seidel J (2009). Conduction at domain walls in oxide multiferroics. Nat. Mater..

[8] Guyonnet J, Gaponenko I, Gariglio S, Paruch P (2011). Ferroelectric materials: Conduction at domain walls in insulating pb(zr0.2ti0.8)o3 thin films (adv. mater. 45/2011). Adv. Mater..

[9] Guyonnet J (2009). Shear effects in lateral piezoresponse force microscopy at $$180^{\circ }$$ ferroelectric domain walls. Appl. Phys. Lett..

[10] Evans DM, Garcia V, Meier D, Bibes M (2020). Domains and domain walls in multiferroics. Phys. Sci. Rev..

[11] Alexe M, Gruverman A (2004). Nanoscale Characterisation of Ferroelectric Materials.

[12] Nataf GF, Guennou M (2020). Optical studies of ferroelectric and ferroelastic domain walls. J. Phys. Condens. Matter.

[13] Haußmann A, Eng LM, Cherifi-Hertel S, Meier D, Seidel J, Gregg M, Ramesh R (2020). Three-dimensional optical analysis of ferroelectric domain walls. Domain Walls.

[14] Yokota H, Uesu Y (2021). Optical second-harmonic generation microscopy as a tool for ferroelastic domain wall exploration. J. Appl. Phys..

[15] Cherifi-Hertel S (2017). Non-Ising and chiral ferroelectric domain walls revealed by nonlinear optical microscopy. Nat. Commun..

[16] Cherifi-Hertel S (2021). Shedding light on non-Ising polar domain walls: Insight from second harmonic generation microscopy and polarimetry analysis. J. Appl. Phys..

[17] Tarrach G (2001). Nanometer spot allocation for Raman spectroscopy on ferroelectrics by polarization and piezoresponse force microscopy. Appl. Phys. Lett..

[18] Shur VY, Zelenovskiy PS (2014). Micro- and nanodomain imaging in uniaxial ferroelectrics: Joint application of optical, confocal Raman, and piezoelectric force microscopy. J. Appl. Phys..

[19] Reitzig S (2021). “Seeing is believing”—In-depth analysis by co-imaging of periodically-poled x-cut lithium niobate thin films. Curr. Comput. Aided Drug Des..

[20] Bonnell DA (2012). Imaging physical phenomena with local probes: From electrons to photons. Rev. Mod. Phys..

[21] Denev SA, Lummen TTA, Barnes E, Kumar A, Gopalan V (2011). Probing ferroelectrics using optical second harmonic generation. J. Am. Ceram. Soc..

[22] Luca GD (2016). Domain wall architecture in tetragonal ferroelectric thin films. Adv. Mater..

[23] Chauleau J-Y, Haltz E, Carrétéro C, Fusil S, Viret M (2017). Multi-stimuli manipulation of antiferromagnetic domains assessed by second-harmonic imaging. Nat. Mater..

[24] Trassin M, Luca GD, Manz S, Fiebig M (2015). Probing ferroelectric domain engineering in BiFeO3thin films by second harmonic generation. Adv. Mater..

[25] Vasudevan RK (2015). Multidimensional dynamic piezoresponse measurements: Unraveling local relaxation behavior in relaxor-ferroelectrics via big data. J. Appl. Phys..

[26] Li L (2018). Machine learning-enabled identification of material phase transitions based on experimental data: Exploring collective dynamics in ferroelectric relaxors. Sci. Adv..

[27] Neumayer SM (2020). To switch or not to switch—A machine learning approach for ferroelectricity. Nanoscale Adv..

[28] Vasudevan RK (2019). Materials science in the artificial intelligence age: High-throughput library generation, machine learning, and a pathway from correlations to the underpinning physics. MRS Commun..

[29] Gaponenko I (2017). Computer vision distortion correction of scanning probe microscopy images. Sci. Rep..

[30] Griffin LA, Gaponenko I, Zhang S, Bassiri-Gharb N (2019). Smart machine learning or discovering meaningful physical and chemical contributions through dimensional stacking. npj Comput. Mater..

[31] Evangelidis G, Psarakis E (2008). Parametric image alignment using enhanced correlation coefficient maximization. IEEE Trans. Pattern Anal. Mach. Intell..

[32] Hastie T, Tibshirani R, Friedman J (2009). The Elements of Statistical Learning.

[33] Griffin LA, Gaponenko I, Bassiri-Gharb N (2020). Better, faster, and less biased machine learning: Electromechanical switching in ferroelectric thin films. Adv. Mater..

[34] Musy, L., Bulanadi, R., Gaponenko, I. & Paruch, P. Hystorian: A processing tool for scanning probe microscopy and other *n*-dimensional dataset. (Submitted). 10.1016/j.ultramic.2021.113345 34214695

[35] Tückmantel P (2021). Local probe comparison of ferroelectric switching event statistics in the creep and depinning regimes in pb(zr0.2ti0.8)o3 thin films. Phys. Rev. Lett..

[36] Weymann, C. *et al.* Non-ising domain walls in *c*-phase ferroelectric lead titanate thin films. (Submitted).

[37] Jo JY (2011). Structural consequences of ferroelectric nanolithography. Nano Lett..

[38] Rousseeuw PJ (1987). Silhouettes: A graphical aid to the interpretation and validation of cluster analysis. J. Comput. Appl. Math..

[39] Musy L, Bulanadi R, Gaponenko I, Paruch P (2021). Hystorian: A processing tool for scanning probe microscopy and other n-dimensional datasets. Ultramicroscopy.

[40] Gaponenko, I., Cherifi-Hertel, S., Acevedo-Salas, U., Bassiri-Gharb, N. & Paruch, P. Correlative imaging of ferroelectric domain walls (2021). 10.26037/yareta:cpcnmarfcvhnlf4ijcfnuvmm2e. 10.1038/s41598-021-04166-y PMC8741908 34997108

