# Supplementary material for: Correlative imaging of ferroelectric domain walls
Source: Sci Rep. 2022 Jan 7;12:165. doi: 10.1038/s41598-021-04166-y (PMC8741908; doi:10.1038/s41598-021-04166-y)
Supplement: Supplementary file 1 — Supplementary Information. [file 41598_2021_4166_MOESM1_ESM.pdf]

Correlative imaging of ferroelectric domain walls: supplementary information

Iaroslav Gaponenko[1,2,*], Salia Cherifi-Hertel[3,+], Ulises Acevedo-Salas[3], Nazanin Bassiri-Gharb[2,4], and Patrycja Paruch[1]

[1]Department of Quantum Matter Physics, University of Geneva, 1211, Geneva, Switzerland
[2]G.W. Woodruff School of Mechanical Engineering, Georgia Institute of Technology, Atlanta, GA, 30332, USA
[3]Université de Strasbourg, CNRS, Institut de Physique et Chimie des Matériaux de Strasbourg, UMR 7504, 67000 Strasbourg, France
[4]School of Materials Science and Engineering, Georgia Institute of Technology, Atlanta, GA, 30332, USA
[*]iaroslav.gaponenko@unige.ch
[+]salia.cherifi@ipcms.unistra.fr

Supplementary Method 1: Complete SHG and SPM dataset

The complete SHG and SPM dataset is shown in Supplementary Figure 1. The acquisition parameters are described in the Methods section in the main manuscript.

SHG intensity images were acquired at intervals of 20° for the 0° and 90° analyzer/polarizer configurations respectively. As can be seen in Supplementary Figure 1(a), there is a large angular variation of the overall intensity of the background - leading to the need for background subtraction prior to analysis, as discussed in the section below.

The SPM scans of the area of interest are shown in Supplementary Figure 1(b). The first line shows the raw topography, piezoresponse phase and piezoresponse amplitude. Prior to their inclusion in the analysis, polynomial subtraction and median offset removal have been applied to the topography and piezoresponse amplitude respectively. This is done in order to avoid the propagation of instrumental or measurement artefacts through the analysis workflow.

Supplementary Discussion 1: Background subtraction

Ferroelectric thin films can exhibit additional SHG contributions due to symmetry breaking at surfaces and interfaces. Here, the studied system consists of a ferroelectric film (PZT 50 nm) grown on a thin buffer electrode (35 nm thick $SrTiO_3$) deposited on the surface of a single crystal ($SrTiO_3(001)$). Given the elongated shape of the photon beam voxel and its micron size, each of these surfaces and interfaces contributes to the overall SHG response. More importantly, surface SHG has a specific symmetry (i.e., an anisotropy that is different from that of the domain walls) and its order of magnitude can be comparable to that of the domain walls in reflection geometry. Eliminating this background signal can thus be indispensable. However, a simple arithmetic background subtraction can represent a delicate task in the case of anisotropic surface SHG response. The left panel of Supplementary Figure 2 shows the background signal measured at the centre of the triangular shaped domains (internal BKG) and the surrounding region (external BKG). While the inner background displays a nearly isotropic response, the outer background shows elongated anisotropy plots. In the artisanal method, the local SHG at the domain wall regions (displayed in Fig. 2(c)) is derived by subtracting the averaged background signal $1/2 \cdot (internal + externalBKG)$) from the intensity collected at the domain wall regions.

The subtraction of the background is also needed in the clustering methods. To do this, the mean polar response is subtracted before clustering, and thus we observe in this case background signatures that are more defined than in the artisanal methods (see Supplementary Figure 2). Furthermore, two distinct external background components showing the same anisotropy but different intensities are revealed in the clustering method. Note that zero-second harmonic emission is expected at c-domains due to the tetragonal structure of $PbZrTiO_3$ in the geometry used for this study (see Methods). The non-zero SHG response observed in these regions can be explained either by surface SHG, as discussed above, or by a misalignment of the local polarization due to local strain or defects (see discussion in the main body text).

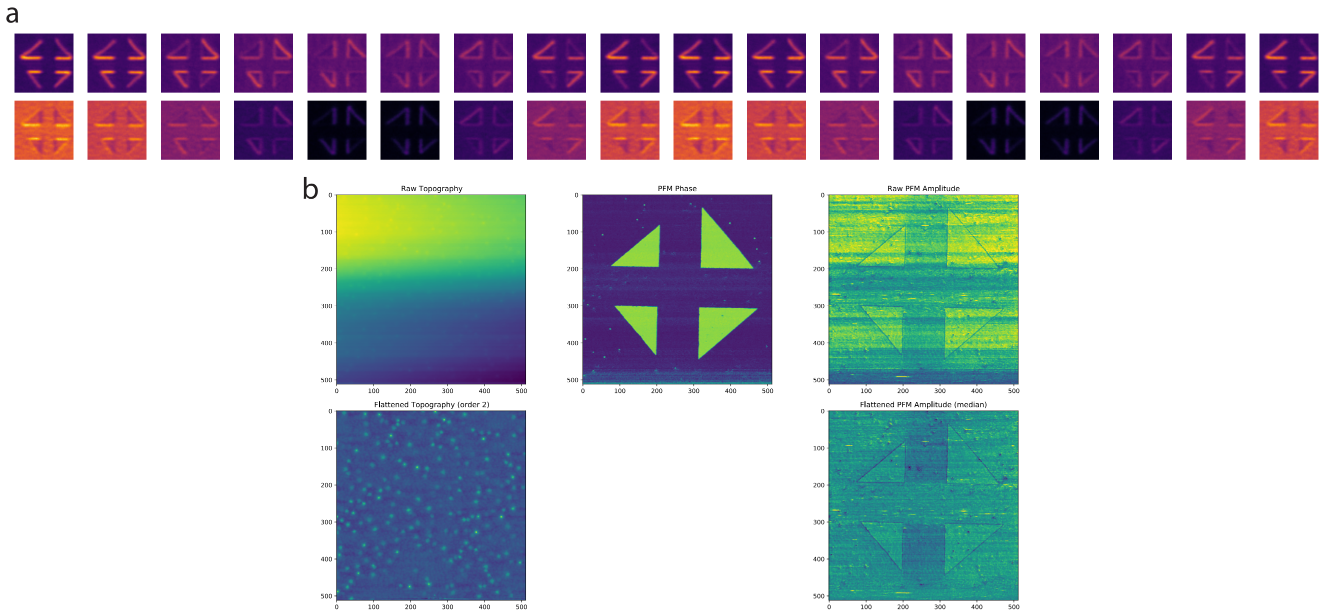

Supplementary Figure 1. Complete SHG and SPM dataset. (a) SHG intensity images were acquired at intervals of 20° for the 0° and 90° analyzer/polarizer configurations respectively. (b) SPM scans of the area of interest. The first line shows the raw topography, piezoresponse phase and piezoresponse amplitude. Prior to their inclusion in the analysis, polynomial subtraction and median offset removal have been applied to the topography and piezoresponse amplitude, respectively.

Supplementary Note 1: K-means clustering

The K-means clustering of the SHG data set is shown for $n = 2$ to $n = 10$ in Supplementary Figure 3. Each clustering is shown with the corresponding cluster map as the leftmost image, and two rows of polar plots. Within each polar plot are two curves, corresponding to the two polarizer angle configurations. The top row of the polar plots shows mean-subtracted and the bottom row shows complete SHG polar plots derived from the corresponding cluster of a given color.

Supplementary Note 2: Silhouette analysis

Silhouette analysis has been performed for K-means clustering with $n = 2$ to $n = 39$. The silhouette plot is shown in Supplementary Figure 4(a), demonstrating a sharp drop in silhouette score at $n = 9$. This can be seen in the corresponding silhouette analysis shown for $n = 2$ to $n = 10$ in Supplementary Figure 4(b-j), with the drop at $n = 9$ giving the most well defined clusters (with a significant amount of data points' individual scores situated above the average silhouette score).

Supplementary Note 3: SHG+SPM Correlated K-means analysis

As the SPM and SHG data sets have been corrected and aligned, it is possible to include the SPM data set within the K-means analysis alongside the SHG. Supplementary Figures 5 and 6 show this for $n = 2$ to $n = 12$ and $n = 13$ to $n = 14$ respectively, with the two data sets given equal weight in the clustering. As can already be seen in Supplementary Figure 5(a), the initial clustering appears to be primarily influenced by the polarization orientation through the piezoresponse force microscopy phase signal. As the number of clusters is increased, however, additional features appear around domain walls, refining the structure more and more until a qualitatively similar distribution of distinct domain wall and background regions appears in Supplementary Figure 6(a) at $n = 13$. As the SPM data are a collection of 1D data points, the mean value of the topographic height, piezoresponse phase and piezoresponse amplitude is extracted for each cluster and written above each set of corresponding polar plots. For instance, one can confirm the qualitative observation in the main text regarding the segregation of the up-polarized domain background signal into two distinct clusters due to the topographic morphology. Indeed, the average height of the two up-background clusters as shown in Supplementary Figure 6(a) and (b) differs significantly.

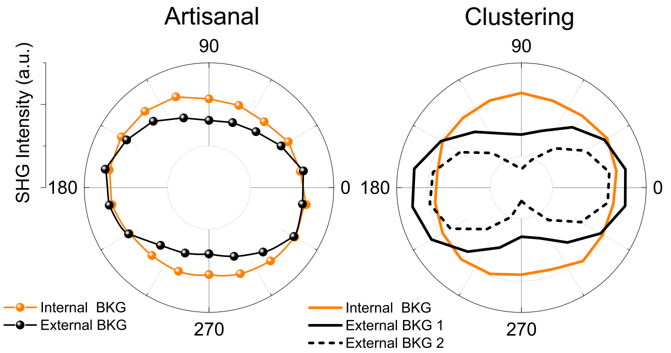

Supplementary Figure 2. Variation of the SHG signal background (BKG) intensity as a function of the analyzer angle at a given fundamental wave polarization ($\varphi = 0^\circ$ in this case). The BKG signal was manually extracted (left) from the centre of triangular shaped c-domains (internal BKG) and in the surrounding region (external background). The corresponding polarimetry response derived from the clustering method is presented in the right panel.

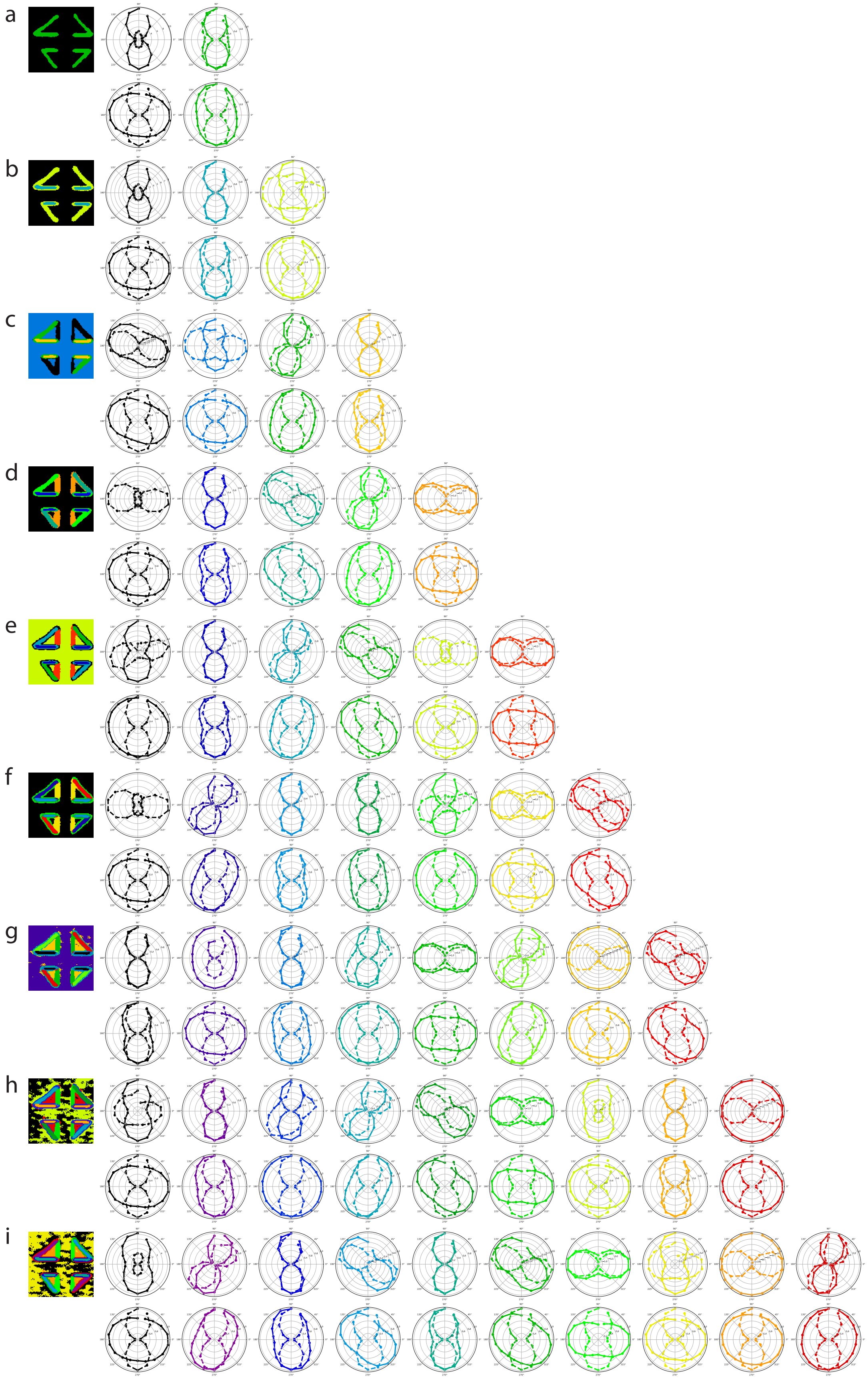

Supplementary Figure 3. K-means clustering for $n = 2$ to $n = 10$. Each clustering is shown with the cluster map as the leftmost image, and two rows of polar plots. The top row shows mean-subtracted and the bottom row shows complete SHG polar plots derived from the corresponding cluster of a given color.

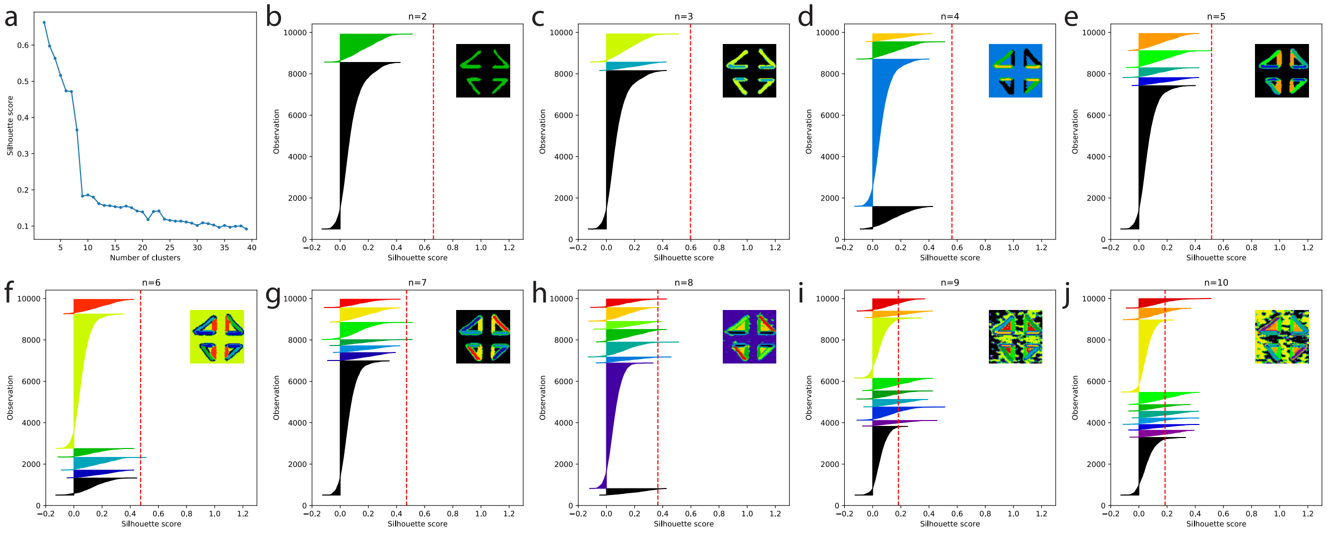

Supplementary Figure 4. Silhouette plot for $n = 2$ to $n = 39$ and individual analysis for the K-means clustering above with $n = 2$ to $n = 10$.

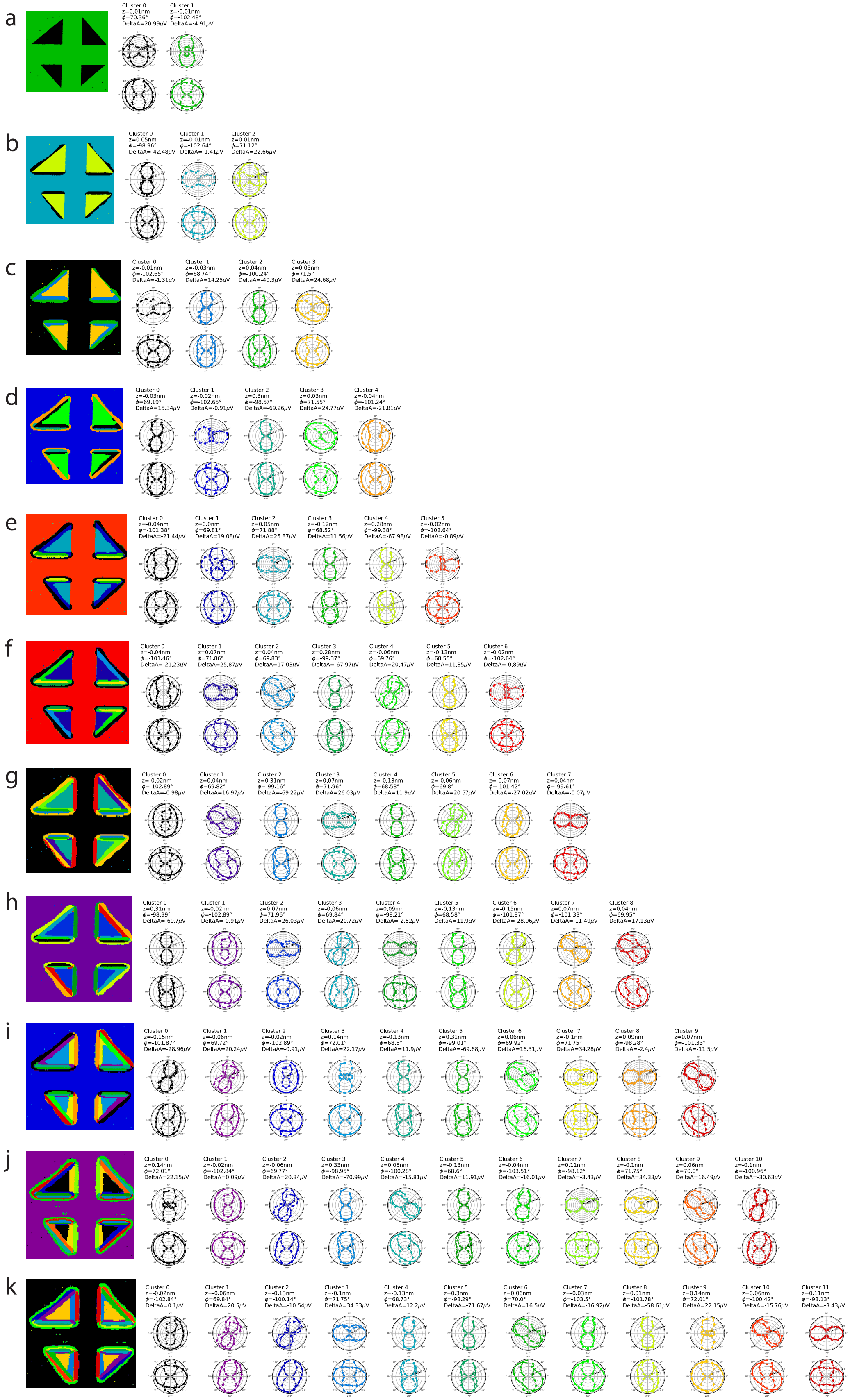

Supplementary Figure 5. K-means clustering for $n = 2$ to $n = 12$ on the combined SPM and SHG dataset. As can be seen even for low cluster numbers, the equal contribution of both data sets favors the polarization orientation discrimination through the piezoresponse phase signal. The topographic height, piezoresponse phase and piezoresponse amplitude are indicated in text for each cluster above the corresponding polar plots (top line mean-subtracted and bottom line raw).

a

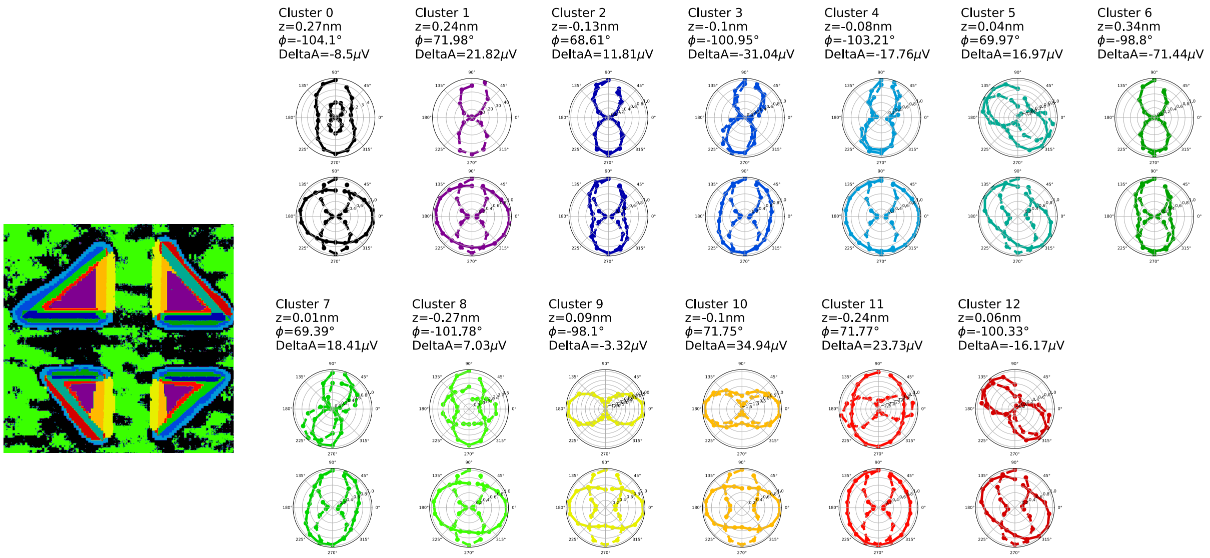

b

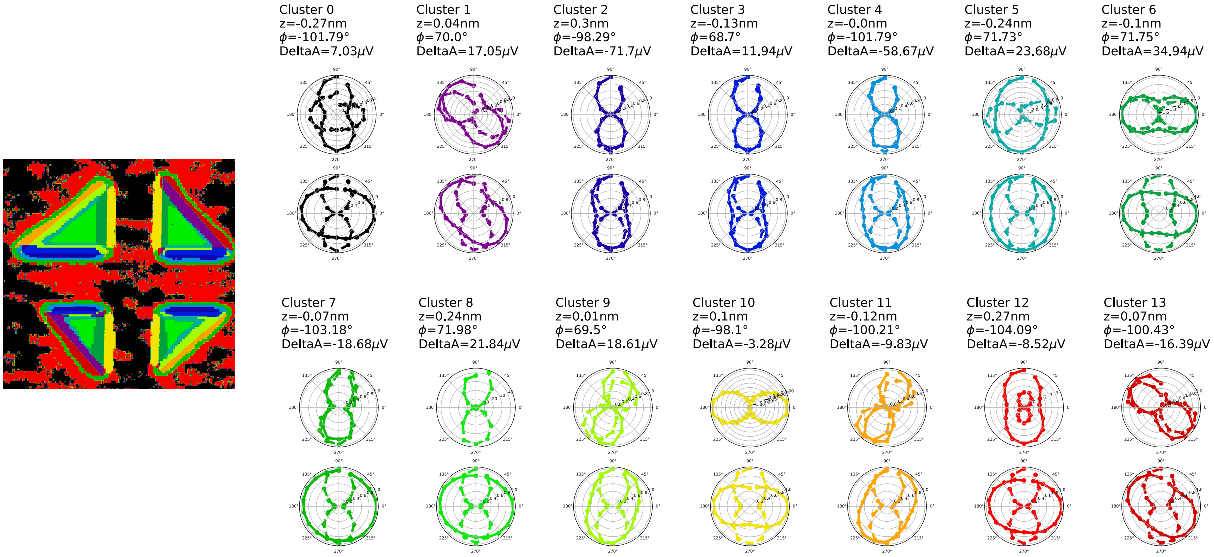

Supplementary Figure 6. K-means clustering for $n = 13$ to $n = 14$ on the combined SPM and SHG dataset. For these high cluster numbers, the background up-polarized region is split into two distinct clusters due to the topographic morphology. The topographic height, piezoresponse phase and piezoresponse amplitude are indicated in text for each cluster above the corresponding polar plots (top line mean-subtracted and bottom line raw).
